# Dynamic enhanced CT: is there a difference between liver metastases of gastroenteropancreatic neuroendocrine tumor and adenocarcinoma

**DOI:** 10.18632/oncotarget.22554

**Published:** 2017-11-20

**Authors:** Yong Cui, Zhong-Wu Li, Xiao-Ting Li, Shun-Yu Gao, Ying Li, Jie Li, Hui-Ci Zhu, Lei Tang, Kun Cao, Ying-Shi Sun

**Affiliations:** ^1^ Department of Radiology, Key Laboratory of Carcinogenesis and Translational Research (Ministry of Education), Peking University Cancer Hospital & Institute, Beijing 100142, China; ^2^ Department of Pathology, Key Laboratory of Carcinogenesis and Translational Research (Ministry of Education), Peking University Cancer Hospital & Institute, Beijing 100142, China; ^3^ Department of Gastrointestinal Oncology, Key Laboratory of Carcinogenesis and Translational Research (Ministry of Education), Peking University Cancer Hospital & Institute, Beijing 100142, China

**Keywords:** gastroenteropancreas, neuroendocrine tumors, adenocarcinomas, computed tomography, differentiation

## Abstract

This study proposed to evaluate the feasibility of dynamic enhanced CT in differentiation of liver metastases of gastroenteropancreatic well-differentiated neuroendocrine tumors (GEP NETs) from GEP adenocarcinomas based on their characteristic features. CT images of 23 well-differentiated (G1 or G2) GEP NETs and 23 GEP adenocarcinomas patients with liver metastases were retrospectively reviewed. The distribution type, shape, intra-tumoral neovascularity, enhancement on hepatic artery phase, dynamic enhancement pattern and lymphadenopathy were subjective analyzed. Meanwhile, the size, number, CT value of tumor and adjacent normal liver parenchyma were measured and the metastasis-to-liver ratios were calculated objectively. Compared with GEP adenocarcinomas, the liver metastases of GEP NETs more frequently demonstrated a hyper enhancement on hepatic artery phase, washout dynamic enhancement pattern, absence of lymphadenopathy and higher metastasis-to-liver ratios on both hepatic artery phase and portal venous phase (*P*=0.017, *P*<0.001, *P* =0.038, *P* <0.001 and *P* =0.008, respectively). Logistic regression analysis showed that the dynamic enhancement pattern (*P*=0.012), and the metastasis-to-liver ratios on hepatic artery phase (*P*=0.009) were independent CT predictors for liver metastases of GEP NETs. The sensitivity and specificity of combing the two predictors in differentiation of liver metastases of GEP adenocarcinomas from GEP NET were 82.6% (19 of 23) and 91.3% (21 of 23), respectively. CT features are helpful in differentiating liver metastases of well-differentiated GEP NETs from that of GEP adenocarcinomas.

## INTRODUCTION

Gastroenteropancreatic neuroendocrine tumors (GEP NETs) arise from the diffuse endocrine system of gastro-intestinal tract and pancreatic islet cells [1]. According to the 2010 WHO classification, GEP NETs involve well-differentiated category tumors (Grade1- Grade 2) with mitoses≤20 and Ki-67≤20% [2]. Some GEP NETs produce peptides causing specific syndromes. But quit a lot of them are non-functioning or synthesizing more than one peptide, mostly are not associated with characteristic hormonal syndromes [3]. Although GEP NETs have low to intermediate pathological grades and exhibit indolent clinical behaviors, they frequently present metastatic disease at diagnosis and the liver is the most involved site [3, 4]. Metastatic NETs show an optimistic long-term outcomes (5-year survival > 50%) after aggressive surgical or effective non-surgical treatment options such as somatostatin analogues, peptide receptor radiotherapy, and intra-arterial therapies [5–7]. Adenocarcinomas of GEP are the most common primary tumor of liver metastases. In patients with liver metastases from GEP adenocarcinomas, acceptable 5-year overall survival only achieved after the resection of colorectal liver metastasis [8]. For the liver metastases of other primary tumor such as gastric cancer or pancreatic cancer, the long-term outcomes are still poor [9]. Therefore, considering that there is a definite survival benefit of local treatments or surgical for metastatic NETs, the differentiation of liver metastases between the GEP NETs and GEP adenocarcinomas may be clinically noteworthy.

As the first line imaging modality, the dynamic enhanced computed tomography (CT) play an important role in the assessment of the stage of GEP NETs. Besides lesion detecting, several previous studies have addressed the values of CT findings, including shape and enhancement features, in the pathological differentiation of GEP NETs [10, 11]. As NETs are commonly hypervascular tumors unlike adenocarcinomas, metastatic NETs may also show hypervascularity on hepatic artery phase CT images. We hypothesized that CT features such as the enhancement degree may be helpful for the differentiation of liver meatastases of GEP NETs from GEP adenocarcinomas. The purpose of our study was to evaluate the value of dynamic enhanced CT features in characterization of difference between liver metastases of GEP NETs and adenocarcinomas.

## RESULTS

**Figure 1:**
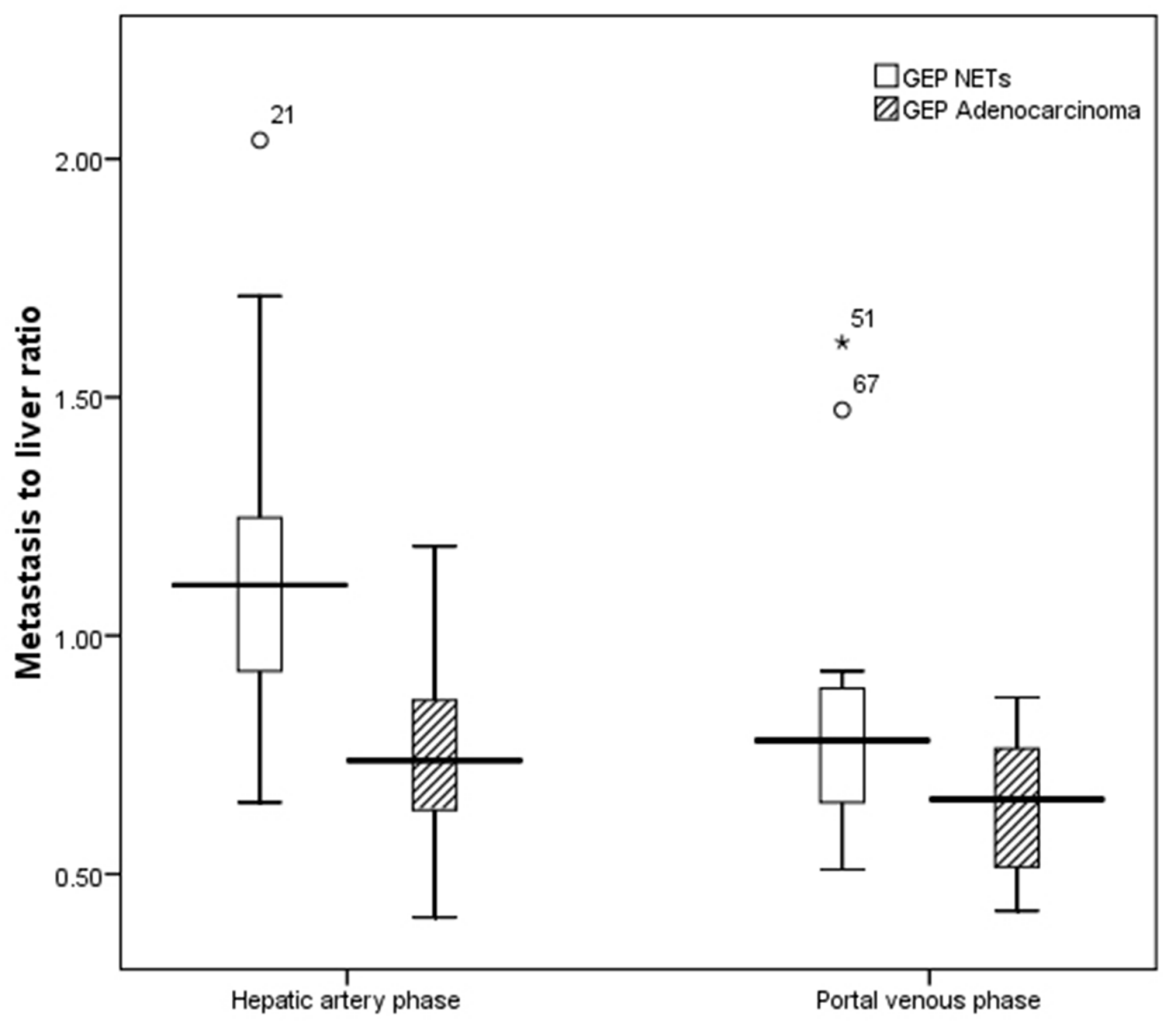
Boxplot of enhancement metastasis to liver ratio between liver metastases of GEP NETs and those from GEP adenocarcinomas Mean metastasis to liver ratio of GEP NETs was significantly greater than that of GEP adenocarcinomas on hepatic artery phase (1.15 vs. 0.77) and portal venous phase (0.81 vs. 0.64).

There was no significantly differences in tumor numbers between patients with liver metastases of GEP NETs (13.48±9.56) and those with GEP adenocarcinomas (12.61±10.30) (Z=-0.472, *P* = 0.637). The mean size of the maximum liver metastases of GEP NETs was 44.57±41.37mm, and that of GEP adenocarcinomas was 50.35±39.40mm. Differences in tumor sizes were not statistically significant (Z=-1.143, *P* = 0.253). Both of the metastasis-to-liver ratios on hepatic artery phase and portal venous phase were higher in the GEP NETs liver metastases (1.15±0.33, 0.81±0.27) compared with GEP adenocarcinomas (0.77±0.20, 0.64±0.15) with statistically significant differences (t=4.774, P<0.001 and t=2.759, *P* =0.008). (Table 1) (Figure 1).

**Table 1:** Comparison of CT Subjective Imaging Features of liver metastases of GEP NETs and GEP adenocarcinomas

Imaging features		NETs	Adenocarcinoma	*χ^2^*	*P*
Distribution type	Restricted	5 (21.7%)	6(78.3%)	0.119	0.73
	Diffuse	18(26.1%)	17(73.9%)		
Shape	Round-oval	23(100.0%)	21(91.3%)	0.523	0.47
	Irregular	0(.0%)	2(8.7%)		
Enhancement on hepatic artery phase	Hyper	17(73.9%)	9(39.1%)	5.662	0.017
	Hypo	6(26.1%)	14(60.9%)		
Dynamic enhancement pattern	Plateau	6(26.1%)	21(91.3%)	-	<0.001
	Washout	15(65.2%)	2(8.7%)		
	Progressive	2(8.7%)	0(0%)		
Lymphadenopathy	Presence	7(30.4%)	14(60.9%)	4.293	0.038
	Absence	16(69.6%)	9(39.1%)		

**Figure 2:**
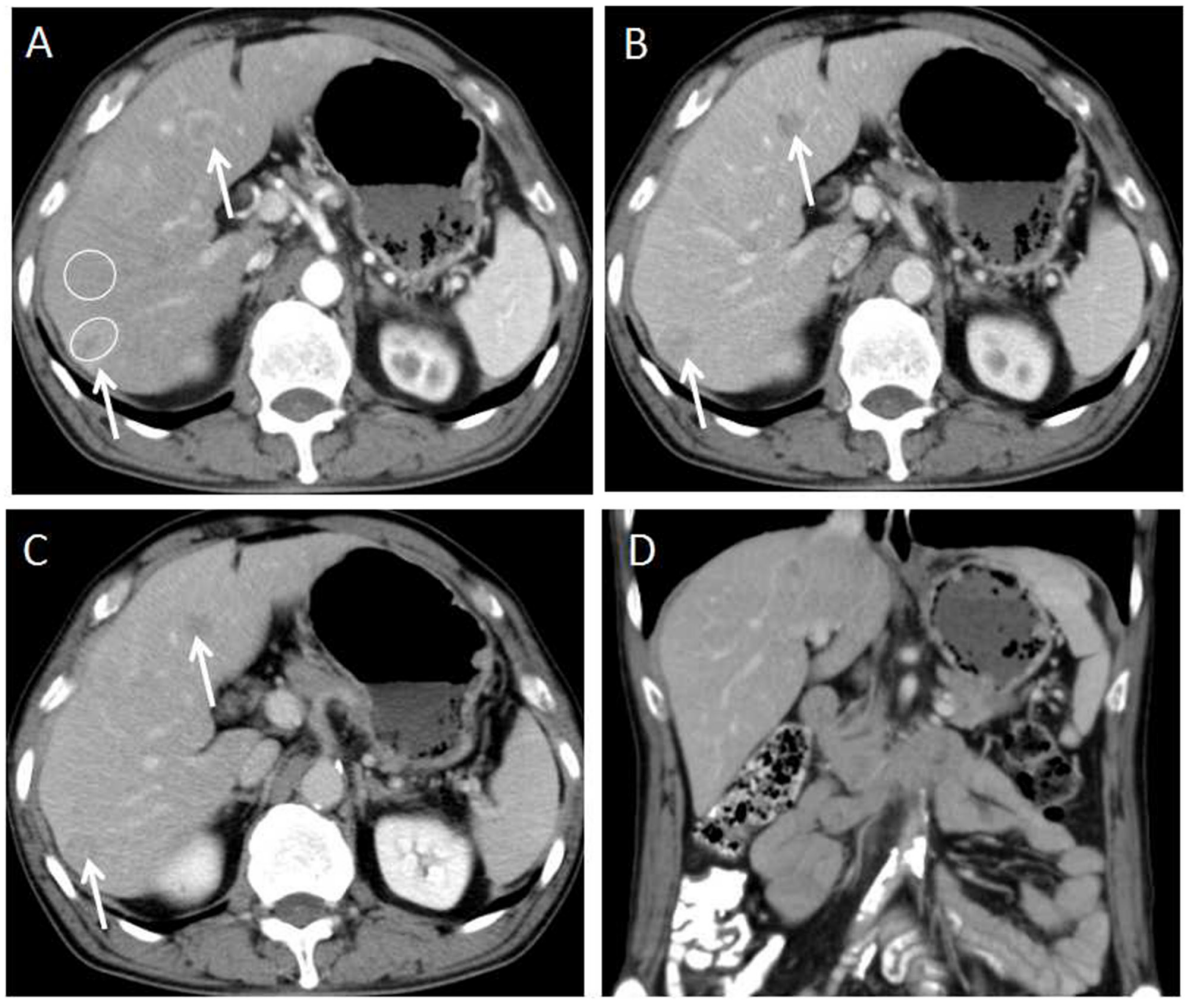
58-year-old male with G2 colon NET **(A)** Axial CT image on hepatic arterial phase demonstrates multiple hyper enhanced liver metastases in the liver(arrows). Two oval shaped ROIs were placed on the largest lesion and adjacent normal liver. The metastasis-to-liver ratios on hepatic artery phase and portal venous phase were 115.7% and 84.8% respectively. **(B, C)** Axial CT images on portal venous phase and equilibrium phase show the liver metastases being hypo enhanced that meets the washout enhancement pattern(arrows). **(D)** Coronal CT image on the portal venous phase shows absent of lymphadenopathy.

**Figure 3:**
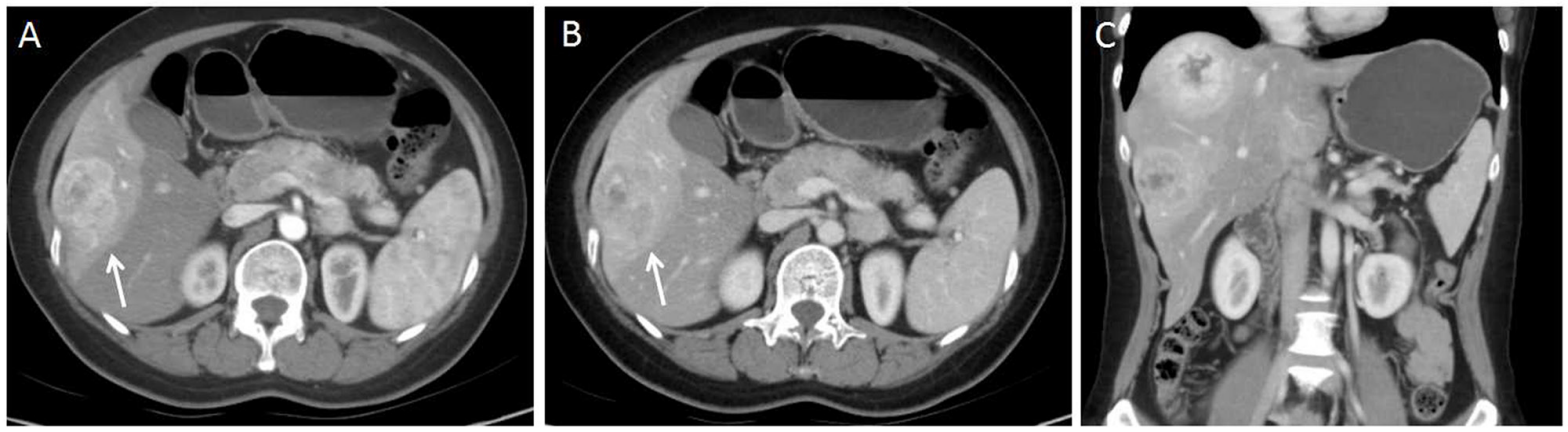
40-year-old woman with G2 pancreas NET **(A)** Axial CT image in the hepatic arterial phase shows a hyper enhanced liver metastasis in the right lobe of liver(arrow). **(B)** On portal venous phase, the lesion demonstrates washout enhancement pattern(arrow). **(C)** No regional or distal lymphadenopathy is present on coronal portal venous phase CT image.

**Figure 4:**
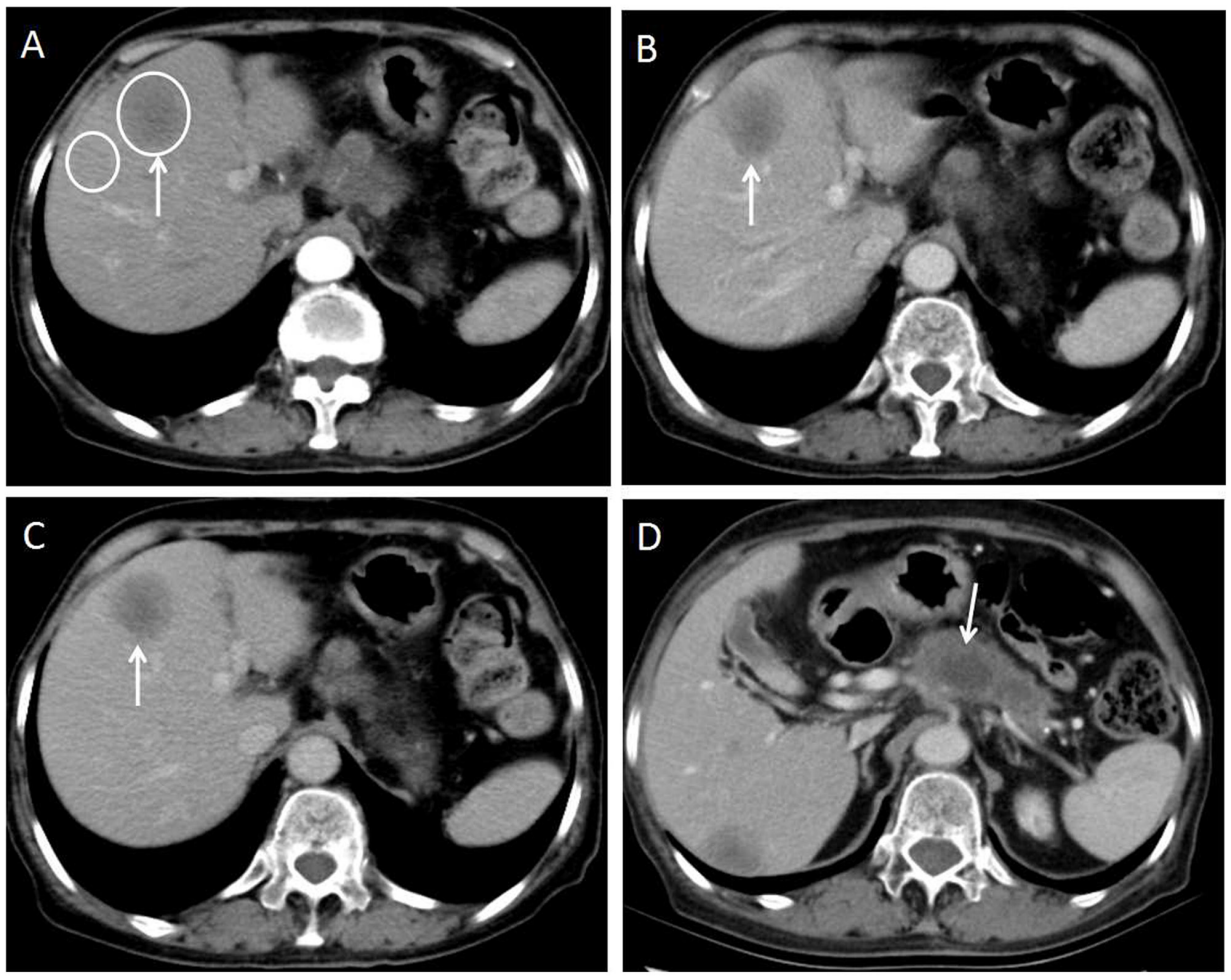
70-year-old woman with moderately-poorly differentiated pancreatic adenocarcinoma **(A)** Axial CT image on the hepatic arterial phase shows a hypo enhanced liver metastasis in the left lobe of the liver(arrow). Two oval shaped ROIs were placed on the largest lesion and adjacent normal liver. The metastasis-to-liver ratios on hepatic artery phase and portal venous phase were 70.7% and 60.2% respectively. **(B and C)** Axial CT image on the portal venous phase and equilibrium phase show the lesion keeps hypo enhancement that meets the plateau enhancement pattern(arrows). **(D)** Axial CT image on the portal venous phase shows hypo enhanced primary tumor in the pancreas(arrow).

**Figure 5:**
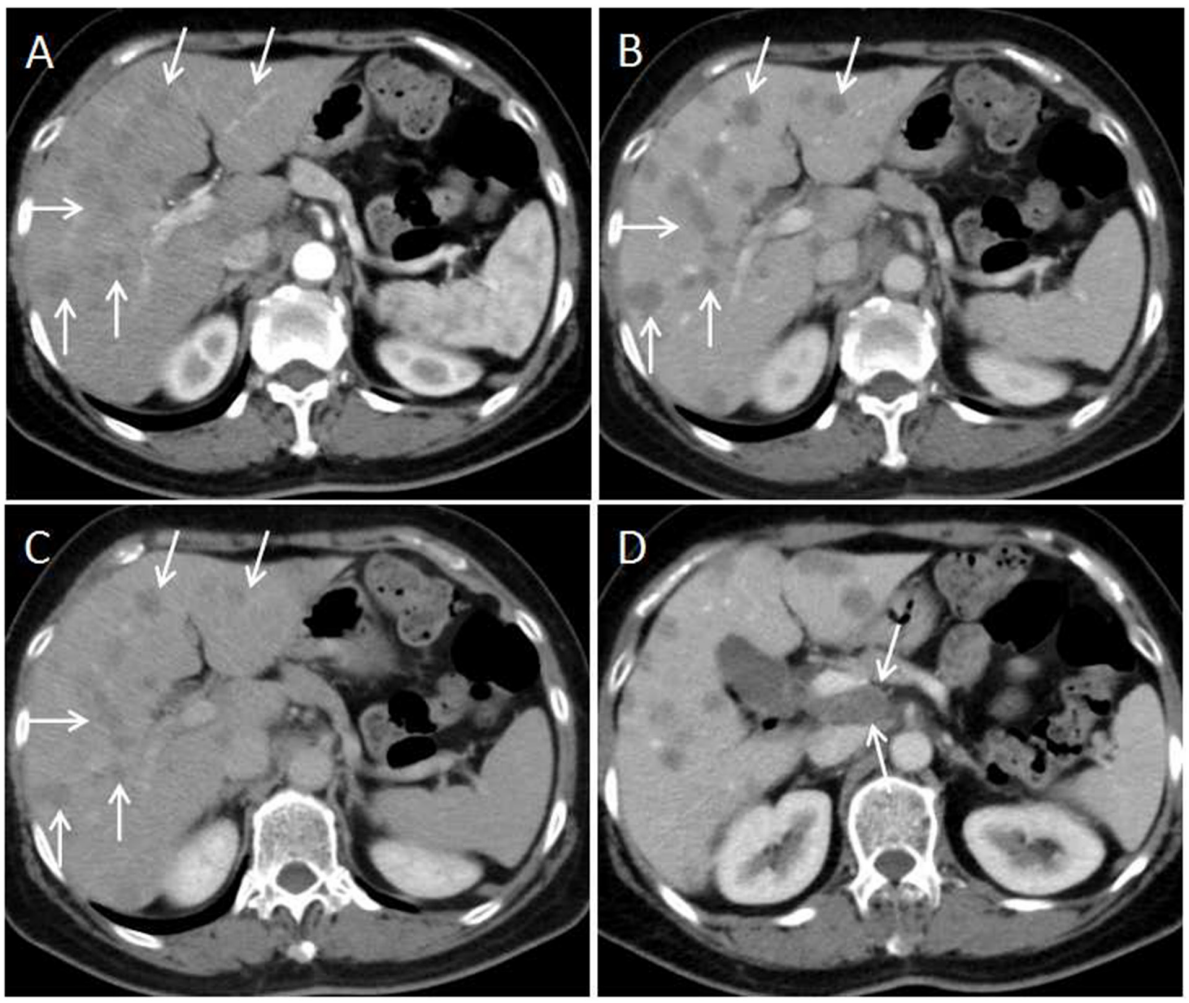
63-year-old woman with moderately differentiated colon adenocarcinoma **(A)** Multiple hypo enhanced liver metastases are presented on hepatic arterial phase CT image(arrows). **(B and C)** On the portal venous phase and equilibrium phase images, the lesions keep hypo enhancement that meets the plateau enhancement pattern(arrows). **(D)** Axial CT image on the portal venous phase presents lymphadenopathy between the portal vein and venae cava inferior (arrows).

For the subjective CT imaging features, the character of enhancement on hepatic artery phase (*P*=0.017), dynamic enhancement pattern (P<0.001) and presence of lymphadenopathy (P=0.038) were significantly different between liver metastases from GEP NETs and from GEP adenocarcinoma (Figures 2-5). The inter-observer variability for subjective analysis ranged from substantial to perfect (for distribution type κ= 0.938, shape κ= 0.789, enhancement on hepatic artery phase κ= 0.775, dynamic enhancement pattern κ= 0.914, and lymphadenopathy κ= 1.000) (Table 1). A perfect ICC was achieved for all objectively measured variables(0.864 for tumor size, 0.919 for CT value of tumor and 0.903 for CT value of adjacent normal liver parenchyma). Finally, the first observer(Y.C.)'s measurement was used for the following analyses.

**Figure 6:**
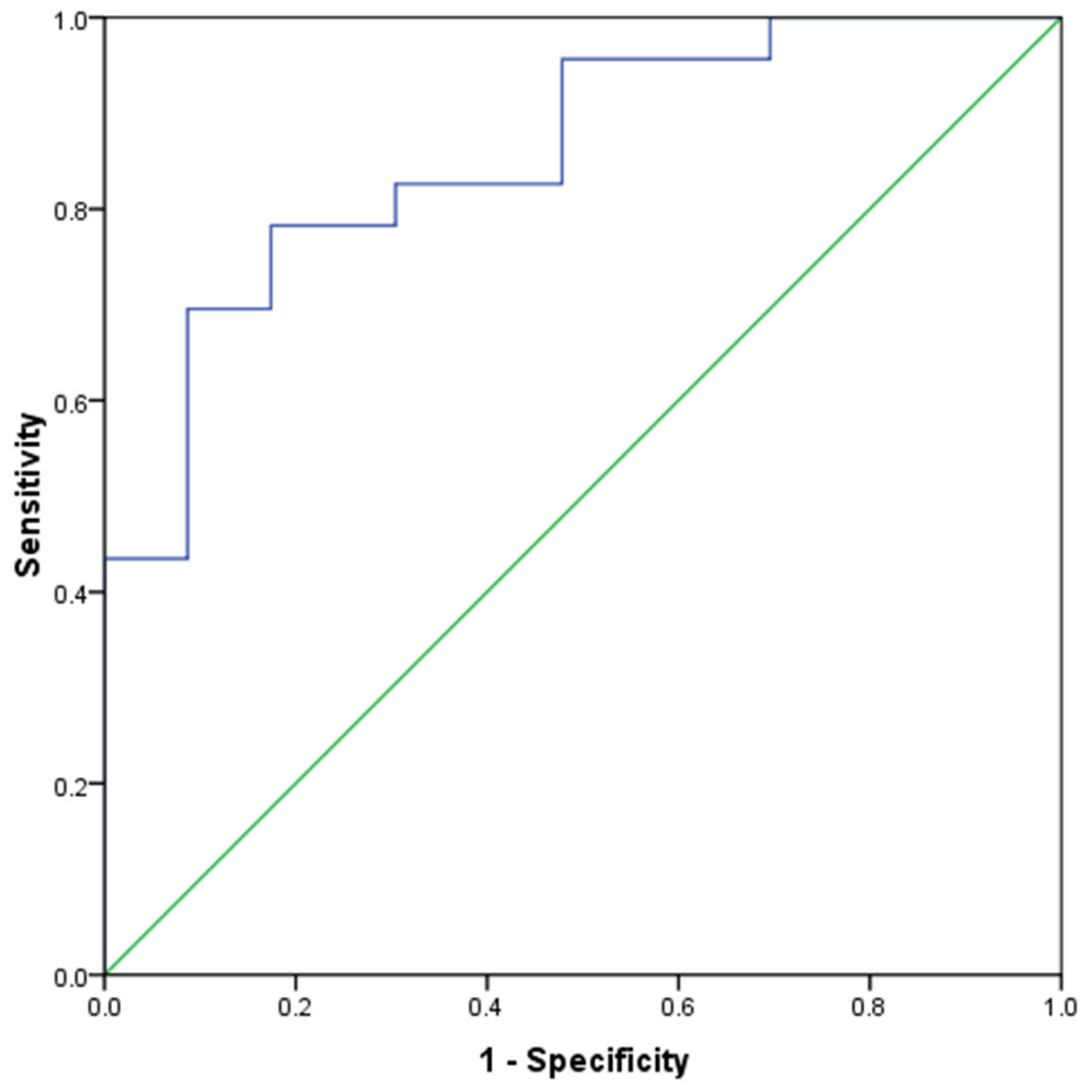
ROC curve of metastasis-to-liver ratios on hepatic artery phase for the prediction of GEP NETs liver metastases The AUROC was 0.856 (95%CI=0.749-0.963). With a cutoff value of 0.898(a value>0.898 indicated GEP NETs and a value≤0.898 indicated GEP adenocarcinomas. The sensitivity and specificity for identifying GEP adenocarcinomas were 82.6%(19 of 23) and 78.3%(18 of 23), respectively.

In addition, binary logistic regression results showed that the dynamic enhancement pattern (Exp (B) = 0.113, 95.0 % CI = 0.020–0.622, *P* = 0.012), the metastasis-to-liver ratios on hepatic artery phase (Exp (B) < 0.001, 95.0 % CI = 0.000–0.067, *P* = 0.009) were independent CT predictors for liver metastases of GEP NETs. In ROC analysis, metastasis-to-liver ratios on hepatic artery phase had an AUC of 0.856 (95%CI=0.749-0.963) in distinguishing GEP adenocarcinomas from GEP NET, with a cutoff value of 0.898(a value>0.898 indicated GEP NETs and a value≤0.898 indicated GEP adenocarcinomas. The sensitivity and specificity for identifying GEP adenocarcinomas were 82.6% (19 of 23) and 78.3% (18 of 23), respectively (Figure 6). And the sensitivity and specificity values of present plateau dynamic enhancement pattern for differentiating liver metastases of GEP adenocarcinomas from GEP NET were 73.9% (17of 23) and 91.3% (21 of 23). When combined the two criterion, the sensitivity and specificity for distinguishing liver metastases of GEP adenocarcinomas from GEP NET were 82.6% (19 of 23) and 91.3% (21 of 23), respectively.

## DISCUSSION

According to the results of the present study, two CT criteria were statistically significant predictors capable of differentiating liver metastases of GEP adenocarcinomas from NETs: present of plateau enhancement dynamic enhancement pattern and a metastasis-to-liver ratios <0.898 on hepatic artery phase. Moreover, when the two features were used in combination, better differentiation of liver metastases of GEP adenocarcinomas from that of NETs could be acheved, with a sensitivity of 82.60% and specificity of 91.30%. This finding indicates that the CT features of liver metastases may be helpful in establishing an accurate diagnosis when distinguishing GEP adenocarcinomas from NETs.

Generally, hyper-enhancement has been considered as the common character of NETs [12, 13]. The liver metastases of NETs tended to present similar enhancement features with their primary lesions [14]. In the study of Kim et al. both the primary gastric NETs and the liver metastases were observed to be hyper-enhanced [15]. For the liver metastases of adenocarcinomas, hypo-enhancement were considered as common features in dynamic enhanced CT or MR scan [16, 17]. Several small series have compared the enhancement of NETs from that of adenocarcinomas,results indicated that higher enhancements were more often found in NETs [10, 15]. Among those studies, no clear result about the divergent imaging features of liver metastases between NETs and adenocarcinomas was presented. To the best of our knowledge, this is the first radiologic report that describing the CT features discrimination of liver metastases of GEP NETs from adenocarcinomas. In our study, the enhancement on hepatic artery phase and the enhancement pattern were significantly different between the NET liver metastases and adenocarcinomas. Liver metastases of GEP NETs were observed to be significantly more hyper-attenuated than those from adenocarcinomas according to subjective analysis, which was also verified on objective analysis. This finding is well in line with our hypothesis and previous studies [18–20] on liver metastases of NETs.

And to the pattern of enhancement, most of liver metastases of GEP NETs demonstrated as washout pattern (15/23), followed by plateau pattern(6/23), contrarialy majority of the liver metastases from adenocarcinomas were plateau pattern(21/23). The pattern of enhancement of liver NETs have been reported in the study of Kim et al. [20], 16 of 34 liver NETs showed enhancing nodule on the hepatic artery phase and washout during the portal and equilibrium phases, and 9 of 34 demonstrated as heterogeneously enhancing nodule on hepatic artery phase, which showed delayed enhancement during the portal and equilibrium phases. Our result well coincided with those investigations that GEP NETs liver metastases showed various enhancement patterns. These findings are believed to be associated with the different degree of fibrosis within the tumors, and that may be the result of combination of vasogenic and fibrogenic activities [21].

In the present study, the present ratio of lymphadenopathy of GEP NETs was lower than that of adenocarcinomas (30.4% vs. 60.9%). The GEP NETs mainly demonstrated a better biology with less lymph node metastases. In the previous studies, lymphadenopathy were found in up to 22.2% of gastric NETs [15], 34.5% of pancreatic NETs [22], and 30.0% in the resectable colorectal NETs [23]. On the contrary, more than 50% of gastric cancer patients have lymph node metastases at diagnosis [24–27]. About 30%–40% lymph nodes of rectal cancer present with invasion [28] and 56% of patients had lymph node metastases in the resectable pancreas adenocarcinomas [29]. It seems that the higher malignancy and stronger invasion of adenocarcinomas can account for this difference.

The present study had some limitations. First, the number of patients in our study was limited by the relatively low incidence of NETs, rendering stratified comparison of patients of different primary location. Second, this was a retrospective study; the start of dynamic enhanced CT used fixed delay time. To eliminate the incoordination of scanning phase, we excluded the cases with insufficient image quality and used enhancement ratio in analysis. Third, pathology result could not be obtained for each of the liver metastases lesions. In some cases, clinical diagnosis were applied, which was also acceptable in previous studies [16]. Our results are promising but should be considered as hypothesis-generating rather than definitive, and prospective studies using novel analysis methods such as radiomics should be conducted in the future.

In conclusion, the subjective and objective features of dynamic enhanced CT are helpful to differentiate liver metastases of well-differentiated GEP NETs from that of GEP adenocarcinomas.

## MATERIALS AND METHODS

### Patients

**Figure 7:**
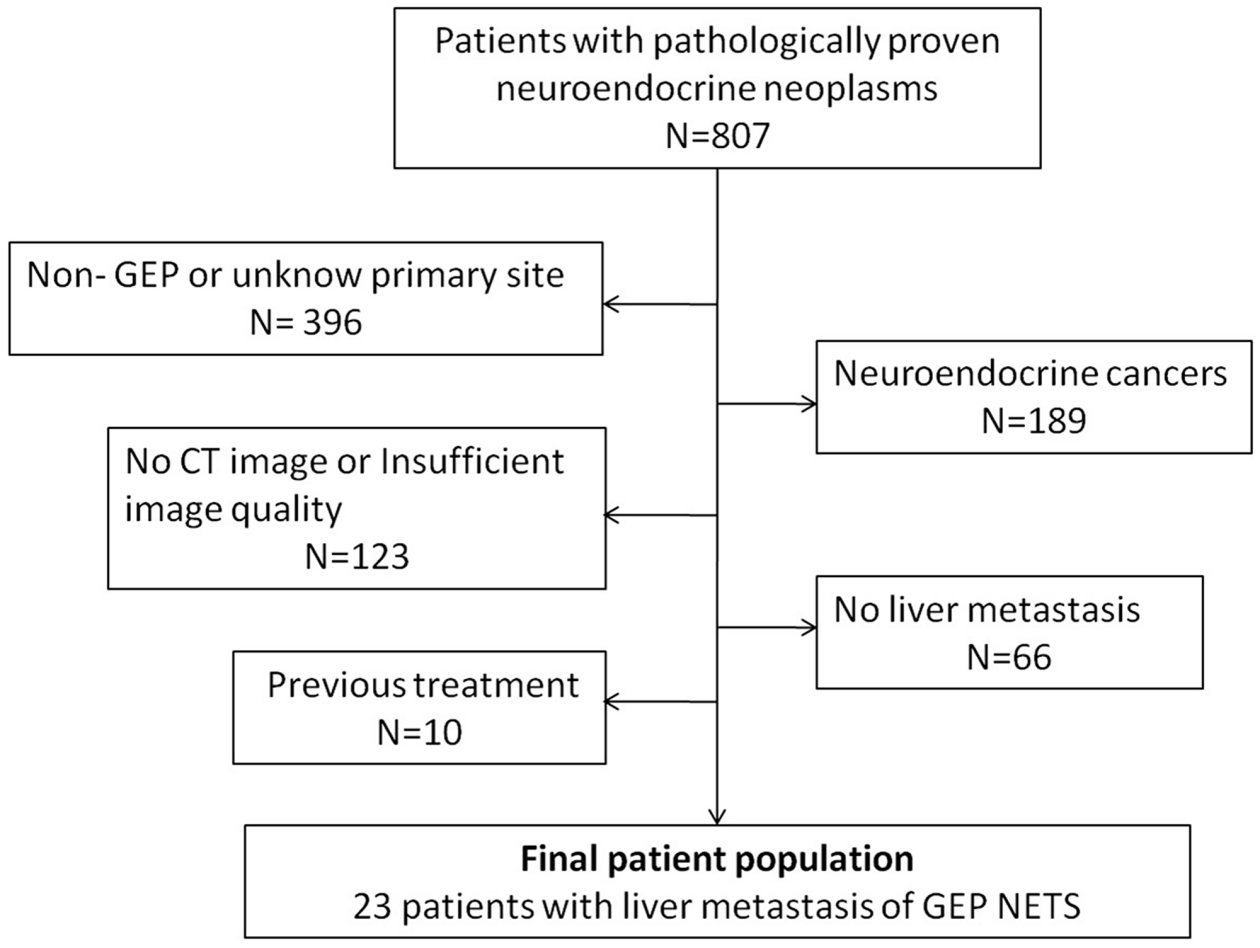
Flowchart of liver metastases patient of GEP NETs inclusion process

This retrospective study was approved by our institutional review board and the requirement for informed consent was waived. Patients’ records of the radiology and pathology departments of our institute from January 2009 through February 2016 were reviewed and cross-referenced. To create a study group of liver metastases of GEP NET, the following inclusion criteria were used: (a) pathological diagnosis of GEP NET, (b) available dynamic enhanced CT images within 4 weeks of pathology, and (c) liver metastases were confirmed on pathology or based on a combination of CT findings and serial imaging or multi-modality imaging such as somatostatin receptor scintigraphy, Gallium-68 positron emission tomography (PET)/CT, determined by a consensus of two experienced abdominal radiologists (Y.C. and S-Y.G., both with more than 15 year experience). In this study, we excluded patients whose primary tumor was not from the GEP, or who had prior treatment before the current CT scan, or insufficient image quality. Finally, 23 patients with pathologically proven GEP NET were enrolled in this study (Figure 7). To serve as the control group, we systemically sampled patients with a diagnosis of GEP adenocarcinomas liver metastases from the patients’ records between the same periods at our institute. These patients fulfilled the criteria: (a) pathological diagnosis of GEP adenocarcinomas, (b) liver metastases was confirmed pathologically or clinically (by using serial image examinations that showed rapid progression of the lesions) and (c) had dynamic enhanced CT images. Patients had treatment before CT exam were excluded. Each patient was given a random number and then sorted by their numbers. Following the order, the first 23 patients matched in term of primary site, age and sex constituted the liver metastases study group of GEP adenocarcinomas. Thus, our retrospective study included 23 patients (13 men,10 women; mean age, 54.43 ±12.34 years; age range, 34-77 years) of liver metastases from GEP NETs and 23 patients (13 men,10 women; mean age, 55.74 ± 9.56 years; age range, 39-74 years) of liver metastases from GEP adenocarcinomas. Each group had the same proportions of primary sites of liver metastases: gastric (n=4), small bowel (n=2), colorectal (n=9), and pancreas (n=8) in each group. For the GEP adenocarcinomas, the pathological grades were well-moderately differentiated in 2 cases, moderately differentiated in 8 cases, moderately-poorly differentiated in 9 cases, and poorly differentiated in 4 cases.

### CT technique

CT was performed with a 64 multidetector CT (MDCT) scanner (LightSpeed VCT or Discovery 750HD, GE Healthcare, Milwaukee, WI, USA) or a 256 MDCT (iCT, Philips Healthcare, Cleveland, USA). Each patient fasted for 6 hours before undergoing CT. After the initial non-enhanced scan, about 100-150 ml (600 mg iodine/kg body weight) of iopromide (Ultravist 300; Schering, Berlin, Germany) or iohexol (Omnipaque 300; Nycomed, Princeton, NJ, USA) through an angiographic catheter inserted into an antecubital vein was administered at a rate of 3 ml/sec by using an automatic power injector. Dynamic enhanced CT scans were obtained 25-30 seconds (hepatic artery phase), 70-75 seconds (portal venous phase) and 150 seconds (equilibrium phase) after the initiation of the intravenous contrast material injection. The scanning range included the upper part of the abdomen from the level of the hepatic dome to the iliac crest. The main scanning parameters of the three scanners were uniform included the following: 120 kVp, automatic tube current modulation, and a tube rotation time of 0.5-0.75 second. Images were displayed in the axial plane with a slice thickness of 5 mm, and multiplanar reformatted images were available for all patients.

### Imaging and pathologic analysis

Subjective image analyses were performed by two experienced abdominal radiologists (Y.C. and S-Y.G., either with more than 15 year experience) using a PACS workstation. Individual reviews were done, and few discrepancies pointed out were resolved by consensus. The radiologists were blinded to patient pathology information. All patients were assessed of the following subjective features: distribution type; lesion shape; intra-tumoral neovascularity; enhancement on hepatic artery phase; dynamic enhancement pattern; and lymphadenopathy were also evaluated. In this study, the distribution type of lesions was described as restricted metastases, which was the metastases confined to one liver lobe or limited to two adjacent segments, or diffuse metastases, that was multifocal lesions located diffuesly in both liver lobes. The lesion shape was assessed as round-oval or irregular. The intra-tumoral neovascularity was defined as the presence of increased, irregular vessels in the tumor. The enhancement on hepatic artery phase was described as hyper enhancing and hypo-enhancing, referencing the adjacent normal liver parenchyma. The dynamic enhancement patterns were recorded as: plateau pattern, the enhancing degree of tumor was similar between the portal venous phase and hepatic artery phase, progressive enhancement pattern, the enhancing degree of tumor on portal venous phase were higher than that of hepatic artery phase, or a washout enhancement pattern, the enhancing degree of tumor on portal venous phase were lower than that of hepatic artery phase. The lymphadenopathy was defined as the presence of lymph node(s) with short axis diameter more than 1cm.

Two observers (Y.C. and S-Y.G.) performed objective measurement independently, including the size, number, CT value of tumor and adjacent normal liver parenchyma. The longest diameter of the biggest metastasis of a patient was measured for the size comparison. The tumor number was counted if liver metastases of a patient less than 20, or else the tumor number of the patient was defined as uncountable. The mean CT value at the maximum level of the biggest liver metastasis and adjacent normal liver parenchyma were measured by using an operator-defined region of interest (ROI), which was specified as a round or oval area including most possible tumor. We took ROI readings from one area of the tumorous lesion and from the other area of adjacent normal liver parenchyma devoid of necrosis or vascular structures. Thereafter, the metastasis-to-liver ratios on hepatic artery phase and portal venous phase were calculated using the following equation: Metastasis-to-liver ratios = CT values of liver metastasis/ CT values of normal liver parenchyma.

All the pathological diagnoses were confirmed by a pathologist (Z-W.L.) with 10 years of experience in gastrointestinal pathology, who was blinded to all clinical information and imaging findings. Pathological tumor grades were determined according to the WHO classification [2] by counting the number of mitoses per 10 high-power fields and the Ki-67 index (percentage of positive cells in the areas of highest nuclear labeling).

### Statistical analysis

The analysis was performed using SPSS software (version 16.0; SPSS, Chicago, IL, USA). The χ^2^ tests or the Fisher's exact test was used for comparison of the subjective interpretation and the independent *t*-test was used to compare the objective measurement between the two groups. Independent factors for differentiating were evaluated using the logistic regression model with backward selection. Receiver operating characteristic (ROC) curves were employed to evaluate the performance of CT features above in the discrimination of the two groups, and area under ROC(AUC) was calculated. An AUC>0.75 suggested clinically useful. The cut-off value was determined by using the maximum Youden's index method. We performed a simple κ analysis to assess inter-observer agreement for subjective variables and calculated intra-class correlation coefficient(ICC) for objective variables. A kappa or ICC of 0.81-1.0, 0.61-0.80, 0.41-0.60, 0.21-0.40 or 0.0-0.20 suggested perfect, substantial, moderate, fair or poor agreement. For all tests, a *P* value <0.05 was considered to indicate statistical significance in all analyses.

## Abbreviations

GEP: Gastroenteropancreatic
NETs: Neuroendocrine tumors
CT: Computed tomography
